# Gründerzeit. Hightech und Alternativen der Wissenschaft in West-Berlin

**DOI:** 10.1007/s00048-022-00352-9

**Published:** 2022-10-27

**Authors:** Max Stadler

**Affiliations:** Bauhaus Universität Weimar, Weimar, Deutschland

**Keywords:** Gegenwissenschaft, Gegenwissen, Wissenschaftspolitik, Informatik, Strukturkrise, West-Berlin, Counter-science, Counter-knowledge, Science policy, Computer science, West-Berlin

## Abstract

Zu den diversen Unternehmungen sozialbewegter „Gegenwissenschaft“, die um 1980 auf der Bildfläche der BRD erschienen, zählte der 1982 gegründete Berliner Wissenschaftsladen e. V., kurz WILAB – eine Art „alternatives“ Spin-off der Technischen Universität Berlin. Der vorliegende Beitrag situiert die Ausgründung des „Ladens“ im Kontext zeitgenössischer Fortschritte der (regionalen) Forschungs- und Technologiepolitik. Gezeigt wird, wie der deindustrialisierenden Inselstadt, qua „innovationspolitischer“ Gegensteuerung, dabei sogar eine gewisse Vorreiterrolle zukam: über die Stadtgrenzen hinaus sichtbare Neuerungen wie die Gründermesse *BIG TECH* oder das 1983 eröffnete *Berliner Innovations- und Gründerzentrum *(BIG), der erste „Incubator“ [sic] der BRD, etwa gingen auf das Konto der 1977/78 lancierten Technologie-Transferstelle der TU Berlin, *TU-transfer*.

Anders gesagt: tendenziell bekam man es hier nun mit Verhältnissen zu tun, die immer weniger mit den Träumen einer „kritischen“, nicht-fremdbestimmten (Gegen‑)Wissenschaft kompatibel waren. Latent konträr zur historiographischen Prominenz des wissenschaftskritischen Zeitgeists fristeten „alternativen“ Zielsetzungen verpflichtete Unternehmungen wie „WILAB“ ein relativ marginalisiertes Nischendasein. Dennoch wirft das am WILAB verfolgte, so gesehen wenig aussichtsreiche Anliegen, eine andere, nämlich „humanere“ Informationstechnologie in die Wege zu leiten, ein instruktives Licht auf die Aufbrüche „unternehmerischer“ Wissenschaft in der BRD um 1980.

## Abstract (en)

Launched in 1982, the so-called *Berliner Wissenschaftsladen e.* *V.* (WILAB) belonged to the scattered West-German ventures in “counter-science”. This article situates the origins of the “Laden” (~ workshop)—an “alternative” spin-off of sorts, spawned from the Technical University of Berlin—in the context of contemporary advances in regional science policy. In this connection, the ailing, de-industrializing “island city” arguably even played a certain pioneering role: elements of its multipronged “innovation offensive”, which peaked in the early-to-mid 1980s, were visible beyond city limits, including the trade show *BIG TECH* and, notably, the *Berlin Center for Innovation and New Enterprises* (BIG), the FRG’s first start-up “incubator”, which opened in 1983.

In other words, politically-minded scientists in tendency now had to deal with conditions that were less and less conducive to dreams of a “socially engaged” and “non-alienated” (counter-)science. Indeed, while hardly opposed to the new gospel of innovation, it’s not surprising that ventures such as WILAB, committed as they were to the production of “socially useful” science, found themselves increasingly marginalized. It’s as such a marginal venture, my argument goes, that WILAB’s prima facie hopeless attempt to initiate a different, more “humane” information technology sheds an instructive light on the emergence of “entrepreneurial” science in the FRG during the 1970s and 1980s.

„Sie haben ein Problem? – Wir helfen mit ungewöhnlichen Methoden! Tel. 7817001.“ Wer Anfang/Mitte der 1980er Jahre die Kreuzberger Fichtestraße entlangspazierte, die- oder derjenige hätte im Schaufenster des Ladengeschäfts mit der Hausnummer 26 ein solches Hinweisschild entdecken können – schräg gegenüber eines lange ausrangierten Gasometers; unweit diverser Szeneeinrichtungen (*Natürlich*: Bioladen und Stehcafé, *Design Dritte Welt*, Frauen-Food-Cooperation und dergleichen); und nicht allzu weit entfernt vom Mehringhof, dem Aushängeschild der West-Berliner Alternativökonomie. Dort, auf dem ehemaligen Betriebsgelände der Schriftgießerei H. Berthold AG, waren unter anderem ansässig: die Zeitschrift *Wechselwirkung. Technik – Naturwissenschaft – Gesellschaft*, Ökotopia GmbH, ein Gesundheitsladen und das „sozialistische Ingenieurskollektiv“ Wuseltronick.

Das Angebot mit den ungewöhnlichen Methoden, das sich an alle richtete, die über Forschung und Wissenschaft nicht im selben Maß wie „Behörden, Großunternehmen [und] Parteien“ verfügen konnten, entstammte ebenfalls diesem Milieu, irgendwo zwischen Neuen Sozialen Bewegungen, „neuer“ Selbstständigkeit und heruntergewirtschafteten Hinterhöfen: der Berliner Wissenschaftsladen e. V., kurz WILAB, eröffnet im April 1982, eine Art Spin-off der TU Berlin, Fachbereich 20 (Informatik). „Gründe sind“, hieß es in einer frühen Selbstdarstellung, „u. a. die Hochschulpolitik des Berliner Senats, das politische Kräfteverhältnis in den Universitäten und die Sparmaßnahmen“ (Beuschel et al. 1983a: 83, 86). Dazu gesellte sich das diffuse „Unbehagen an Technik und Wissenschaft“ jener Jahre, das offenbar auch an der TU Berlin nicht ganz spurlos vorüberging. „[W]ie komme ich mit dem, was ich mache“, stand im Gründungsmanifest, „aus der Isolierung des Elfenbeinturms heraus?“ (Bickenbach & Keil 1981: 2). Der Dunstkreis des Projekts „WILAB“ wurde dort gleich mitbenannt: diverse Initiativen in Sachen „kritischer“, „alternativer“, „sozialer“ und/oder „emanzipativer“ Wissenschaft, die sich in jüngster Zeit häuften. Darunter: das Freiburger Ökoinstitut, allerlei Unternehmungen in Sachen „angepasste Technologien“, die Forschungs- und Beratungsstelle Informationstechnologie (FORBIT) in Hamburg, überhaupt „Läden“ (Frauen‑, Kinder‑, Mieterläden) sowie, als unmittelbares Vorbild, die *Wetenschapswinkels* in den Niederlanden.

Auch das Projekt „WILAB“, angetreten mit dem Ziel, eine „Brücke zwischen sozialer Bewegung und Wissenschaft“ zu bilden (Beuschel et al. 1983b), war entsprechend vom Geist beseelt, alles irgendwie* anders* zu machen – ein Unterfangen, das selbst im anvisierten Kundenkreis nicht überall auf Gegenliebe stieß. „[O]b und unter welchen Bedingungen Wissenschaftler als Wissenschaftler den sozialen Bewegungen nutzen können“, wäre zumindest eine offene Frage, sinnierte ob der damaligen Konjunktur von „Gegen-Wissenschaft“ nicht nur *Chips & Kabel*, der „Medienrundbrief“ der Grünen … und sei’s nur aufgrund der prinzipiell „systemerhaltende[n] Funktion von Wissenschaft“ (Quandel & Schmahl 1985: 7–8). Ähnliche Bedenken plagten die Protagonist*innen derartiger Unternehmungen selbst. „Mein Dilemma besteht darin“, so drang es aus deren Reihen, „daß ich außerhalb meiner hauptberuflichen Tätigkeit an der TU [Berlin] die Auswirkungen dessen bekämpfe, wofür ich durch meine Arbeit an der TU zum Teil mitverantwortlich bin“ (Keil-Slawik 1985: i). Als Unterfangen stieß das Ganze in jedem Fall schnell an definitive Grenzen. Zeit‑, Kapazitäts- und Grundsatzfragen gehörten, wenig überraschend, zur Tagesordnung. Und auch die Tatsache, dass der durchschnittliche Wissenschaftsladen – ob nun in Berlin, Köln oder Göttingen – einem „Milchgeschäft ähnlicher [sah] als einer der üblichen in Beton gehaltenen akademischen Brutstätten“ (Wedemeyer 1984: 41), hatte einen schlichten Grund: „Ethos ist doch hier [in der Wissenschaft] money“ (Siebel et al. 1982: 38).

Diesen eher bescheidenen Verhältnissen zum Trotz soll im Folgenden die Karriere des WILAB skizziert werden – der Fall eignet sich gut als Parabel auf die Umstände, unter denen Projekte der „Gegenwissenschaft“ damals aufblühten, ohne allerdings nachhaltig institutionell Fuß fassen zu können. Hervorzuheben gilt es hier Letzteres, insofern der Verdacht der Komplizenschaft auf den ersten Blick durchaus naheliegt: er zirkulierte bereits damals – handelte es sich bei all der gegenwissenschaftlichen Betriebsamkeit nicht doch nur um „scheinpolitische Inselchen“ im Einklang mit den „spätkapitalistischen Erfordernissen“? (Brockner 1985: 8). Und mit allgemeineren historiographischen Tendenzen liegt er auf Linie. So findet sich der Topos in der eher überschaubaren Selbsthistorisierung der „WILA-Bewegung“ („WILAs“ fanden sich auch in anderen Universitätsstädten).^1^ Vor allem aber weht er zumindest durch Teile der mittlerweile recht üppigen Literatur zur linksalternativen Projektemacherei: deren theoretische Allüren sowie antibürokratische, antistaatliche und (klein)bürgerliche Impulse sehen sich heute schnell mal dem Vorwurf ausgesetzt, die vermeintliche Verflüssigung der Verhältnisse mit in die Wege geleitet zu haben.^2^ Supplementiert wird dieses Motiv in jüngster Zeit – Stichwort: alternative Fakten – mit einer Art epistemologischen Verfallserzählung, die um die bereits zeitgenössisch bemühte Diagnose sozialbewegter „Irrationalität“ als eine ihrer Urszenen kreist (vgl. Reichardt 2021; Sarasin 2019; Speit 2021).^3^ Nun soll beziehungsweise kann hier gar nicht in Abrede gestellt werden, dass derartige Linien in die ohnehin äußerst heterogenen Milieus des alternativen Spektrums gezogen werden können. Dass andere Lesarten aber möglich sind und die Sache allermindestens komplizierter liegt – zumal mit Blick auf „Wissenschaft“ –, lässt sich selbst an einem eher marginalen Fallbeispiel wie „WILAB“ durchspielen.

Zwar konvergierte, wie sich zeigen wird, auch der am WILAB zu Tage tretende Wille zum „Transfer“ durchaus mit den besagten spätkapitalistischen Verwerfungen des akademischen Sektors, die vonseiten der BRD-Zeitgeschichte heute vermehrt unter Schlagworten wie Ökonomisierung, Managerialisierung oder Neoliberalisierung thematisiert werden.^4^ Und auch am WILAB bildete das epistemologische Koordinatensystem, das die Historiographie in Sachen Bewegungen tendenziell in Anschlag bringt – Betroffenheit, Ganzheitlichkeit, Emotionalität – den Horizont „kritischer“ Wissenschaft.^5^ Quasi im Gegenzug entfernte man sich dort vom eingeschliffenen Diskurs von wegen „imperialistischer Expansion“ (via Schlüsseltechnologien) und ähnlichem, wie man dies der Anti-EDV-Broschüre mit dem Titel *Technische Universität Berlin: Forschungsstätte der Bourgeoisie* typischerweise noch hätte entnehmen können (Zelle Kybernetik 1976). Was nun aber noch nicht heißt, dass der kritische Impetus der WILA-Projektemacher deshalb schon mit der neuerlichen Betriebsamkeit des (ebenfalls kaum monolithischen) „Establishments“ identisch wäre. Im Gegenteil: Wie ich zeigen möchte, entpuppen sich im Kontext der zeitgenössischen West-Berliner Innovationspolitik, die als „Modell Berlin“ in diesem Zusammenhang durchaus eine gewisse Vorreiterrolle für sich reklamieren konnte (vgl. Burmeister & Canzler 1988), WILAB und ähnlich gepolte para-akademische Unternehmungen weniger als Anfang vom Ende, denn als Schwundstufen universitärer Reformbestrebungen der 1970er Jahre (vgl. dazu etwa Rudloff 2018; Scholz 2019). Auch solche institutionellen Dynamiken, so mein Argument, gilt es in punkto „Gegenwissen“ im Blick zu behalten: denn tendenziell bekam man es hier nun mit Verhältnissen zu tun, die immer weniger mit den Träumen einer (wie auch immer) „kritischen“, nicht-fremdbestimmten Wissenschaft kompatibel waren. Nicht ganz zufällig, so wird sich zeigen, rekrutierte sich die Stammbelegschaft des WILAB aus dem Umfeld des erwähnten FB 20: des „weltweit“ größten Informatikfachbereichs, wie man sich damals brüstete (Beuschel et al. 1980: 20). Umgekehrt wirft das am WILAB verfolgte, wenig aussichtsreiche Anliegen, auf diesem Weg eine *andere*, nämlich „humanere“ Informationstechnologie in die Wege zu leiten – ein Anliegen, das das Projekt „WILAB“ mit zeitgenössischen Experimenten in Sachen „nützliche Produkte“, „alternative Technologien“ und „partizipatives“ Design verband (Asaro 2000; Quet 2013; Stewart-Halevy 2021) –, so ein durchaus instruktives Licht auf die Aufbrüche „unternehmerischer“ Wissenschaft in der BRD um 1980.

Durchgeklungen war ja schon, dass Hochschulpolitik, Sparmaßnahmen und entsprechend neusortierte „Kräfteverhältnisse“ mit an der Wiege des Kreuzberger WILAB standen. Es kommt mir im Weiteren also weniger auf das Phänomen „WILA“ als solchem an, noch unbedingt auf das Tagein-tagaus der Ladenbetreiber*innen. Was hier in erster Linie interessiert, ist der Versuch, das Projekt „WILAB“ als Symptom West-Berliner wissenschaftspolitischer Verhältnisse um 1980 zu verorten. Kaum überraschend bewegten sich diese damals nämlich recht allgemein in Richtung „Überwindung“ des Elfenbeinturms: Je weniger dort vom Glanz der einstigen „Elektropolis“ zu spüren war – *Is Berlin a Dying City?, Massenentlassung bei AEG!* und ähnlich lautende Schlagzeilen begannen sich in den 1970er Jahren zu häufen – desto intensiver florierten auch die Fluchtversuche nach vorn. Zum Krisensoundtrack der deindustrialisierenden Frontstadt mischten sich infolge betont hoffnungsvollere Slogans, die für das Verständnis gegenwissenschaftlicher Umtriebe dann auch nicht weniger relevant sind als die Schnittmengen mit dem alternativen Spektrum: „Innovationen statt Subventionen“, die TU als „Dienstleistungsbetrieb Berlins“, überhaupt Berlin als „Problemlösungslabor“ und als „Stadt der Erfinder und Gründer“, gar ein „Hightech-Mekka“ an der Spree sahen manche schon am Horizont heraufziehen (siehe u. a. Burmeister & Canzler 1988: 55; Huncke 1987: 3; Berger 1978). Ein „Schaufenster der Wissenschaft“ unterhielt die Berliner TU zwischenzeitlich denn auch ganz offiziell – bezeichnenderweise im KaDeWe (Anonym 1985b).

Ebenfalls wenig überraschend wurden im Zuge entsprechender städtischer Reanimationsversuche qua „Innovationspolitik“ nicht in erster Linie „kritische“ Zielsetzungen verfolgt. Vielmehr waren es solche, die, sehr grob vereinfachend gesagt, um „money“ kreisten – und um das damals noch einigermaßen neuartige Vokabular von „Innovation“, „Transfer“, „Spin-offs“ und dergleichen.^6^ Diesbezüglich immerhin konnte sich die, wie böse Zungen behaupteten, zum „Industriemuseum“ verkommene Stadt aber bald durchaus sehen lassen (DGB Landesbezirk Berlin 1984: 42). Galten die West-Berliner Universitäten noch vor wenigen Jahren als „Schmuddelkinder der Nation“, wie das (kurzlebige) Alternativmagazin *Kassandra *der TU Berlin 1984 notierte, so gäben sich dort nun „Industrielle […] auf den Festbanketten die Klinken in die Hand“, um derart die „Privatisierung von Wissenschaft und Forschung“ voranzutreiben (Heymann 1984: 55). Der* Spiegel* wollte immerhin von einer „inflationären Neugründungseuphorie“ in deren Dunstkreis wissen (Anonym 1988: 91). Und von „neuen Gründerjahre[n] in Berlin“ schwärmte nicht zuletzt *TU-transfer*, die 1977/78 lancierte Technologietransferstelle der TU (Allesch 1984: 407).

Dass derartigen Visionen und entsprechenden Initiativen – jenen „von oben“, wenn man so will – im Weiteren ebenfalls ein gewisser Raum gegeben wird, hat demnach einen simplen Grund: die Form von „Gegenwissenschaft“, die an Orten wie WILAB kultiviert werden sollte, lässt sich schlecht verstehen, reduziert man sie auf Kritik und Protest. Die Verschränkung von post-industrieller Malaise und Hightech-Hoffnungsschimmer schlug bekanntlich nicht zuletzt auf die Universitäten durch, die, wie bereits angedeutet, sich nun ihrerseits mit neuartigen Erwartungen konfrontiert sahen – und diese schürten. „Mit […] ihrem immer unverhüllteren Ausverkauf an das Industriekapital (durch Technologietransferstellen, Wissenschaftsparks, Gründerzentralen, Stiftungslehrstühle, Mikroelektronik- und Gen-Zentren) […] werden überzeugende Alternativen und Gegenstrategien lebensnotwendig“, hieß es dann auch in den dramatischeren Auslassungen zum Thema „Wissenschaftsläden“ (Frerks & Heinrichs 1985: 645). Und wie dem auch sei: der Blick auf die Frontverläufe der „neuen“ Berliner Gründerjahre – Produkt, wie noch deutlicher werden wird, der damaligen Melange aus „sozialliberalen“ und „neokonservativen“ Fortschritten der Wissenschaftspolitik –, zeigt, dass der derart sich manifestierende staatliche Gestaltungswille, der gerne als Frage von „Autonomie“ versus „verplante Forschung“ und ähnlichen Formeln verhandelt wurde und wird (vgl. Leendertz 2013; Stadler & Wulz 2020), in den widerspenstigen Milieus der Wissenschaft durchaus anders konnotiert daherkommen konnte. Dort wurde in der Regel weniger an der eingeforderten Verwertbarkeit universitärer Forschung per se Anstoß genommen, als an deren asymmetrischer „Privatisierung“ – unter anderem deshalb ja das Angebot mit den „ungewöhnlichen Methoden“.

Notwendigerweise bewegte sich der WILAB damit auf einem schmalen Grat: irgendwo zwischen „Ablehnung“ und „Aneignung“ von (Hoch‑)Technologien, deren zunehmend multinationale Dispersion und massive Forschungsintensität dem Traum von der „alternativen“ Technologie auch strukturell entgegenlief. Es liegt in der Natur der Sache, dass sich dies alles anhand eines Kreuzberger TU-Außenpostens nur schematisch thematisieren lässt; immerhin überlagern sich dort, wie bereits deutlich geworden sein sollte, einige relevante historische Fäden. So stellte das Antlitz der „Deindustrialisierung“ nicht nur die Szenerie (Leerstand, Hinterhöfe, „Läden“), die damit angesprochenen Dynamiken lieferten auch reichlich Material für „betroffenenorientierte“ Forschung – Rationalisierung, Arbeitsintensivierung, Forcierung von „neuen“ Technologien. Das passierte damals zwar nicht nur, aber offenbar auch nicht zuletzt in der „Inselstadt“: jener „verlängerten Werkbank der westdeutschen Industrie“, der zwischen 1960 und 1989 an die 130.000 industrielle Arbeitsplätze abhanden kamen (Ohe 1981: 87ff).^7^ Insbesondere die TU hätte „ihre Möglichkeiten, aktiv an der Stadtentwicklung und Regionalpolitik auf dem Gebiet der Wirtschaftsförderung mitzuwirken, [deshalb] wesentlich ausgebaut“, wie der TU-Präsident Jürgen Starnick (im Amt: 1979–1985) gerne und oft betonte (Starnick 1982: 3). So etwa mietete die TU damals höchstselbst 30.000 m^2^ neuen Leerstand an – die ehemalige AEG-Fabrik in der Ackerstraße (im Wedding) –, nur um dort, wie kritische Geister glaubten, eine Art „Wissenschaftsladen von Kapitalisten für Kapitalisten“ zu betreiben (Brockner 1985: 7). (Gemeint war das 1983 eröffnete *Berliner Innovations- und Gründerzentrum*, BIG: dazu unten also mehr). „Thinking globally, acting locally“, hieß es auch am 1982 eröffneten WILAB, ob es nun um Beistand bei der Einführung neuer EDV-Systeme ging oder – bis Mitte 1984 waren bereits knapp 250 Kundengesuche eingegangen – eine „Stellungnahme gegen […] Tiefkühlkost in einer Kindertagesstätte“ (Anonym 1982a: 53; WILAB 1983: 25). Als Vorgeschichte dazu skizziert Teil 1 den sich aufbauenden Druck in Richtung „bedarfsorientierte“ Wissenschaft, der in den 1970er Jahren auch West-Berliner Wissenschaftler*innen einholte. Teil 2 handelt von der in diesem Kontext formannehmenden „Innovationspolitik“ beziehungsweise deren Instrumenten, darunter das erwähnte Gründerzentrum, BIG. Teil 3 verortet die Genese des WILAB vis-à-vis solcher, zeitgenössischen Bemühungen um „Wissenschaftstransfer“. Und schließlich, eher als Ausblick, widmet sich Teil 4 dem damit verknüpften Projekt „kritische Informatik“ – ein Projekt, das in den 1980er Jahren, zwischen Pershing-Raketen und *Strategic Defense Initiative *(SDI), noch einmal an Dringlichkeit gewann.

## Forschen, wo Probleme sind

Als sich im Laufe des Jahres 1979 fünf „Assistenten und Assistentinnen“ des FB 20 um das vorläufige Akronym PROWILA („Projekt Wissenschaftsladen“) zusammenfanden, lagen einige dieser Dinge noch in düsterer Zukunft – zumal der Trubel um SDI („Star Wars“), der nicht nur Informatiker*innen im größeren Stil mobilisieren sollte.

Man musste aber gar nicht in die Sterne greifen. Der unmittelbare Anlass lag, wie erwähnt, vor der Haustür: „etwas gegen die Auswirkungen der neuen Hochschulgesetze […] unternehmen“ wollte man (Bickenbach & Keil 1981: 41). Gemeint war die Hochschulrahmengesetz (HRG)-Novellierung 1976, deren Um- beziehungsweise „Durchsetzung“ Studierende zurück auf die Straßen brachte. Die diesbezüglichen Reizworte lauteten: Regelstudienzeit, Zwischenprüfungen („Auslese“), Rückbau von „Mitbestimmung“ (auch für den Mittelbau), dazu BAföG-Kürzungen wie überhaupt „Entschlackung“. „Stück für Stück“ würden die Errungenschaften der Reformjahre nun „demontiert“, notierte man bei PROWILA. „Übrig blieb[e] eine den modernen Industrieerfordernissen angepaßte und durchrationalisierte […] Universität“ (Bickenbach & Keil 1981: 42). Die erste verbriefte Aktion der Ladengründer *in-the-making *wiederum bestand darin, der ebenfalls noch jungen *taz*-Redaktion einen Besuch abzustatten. Dort hatte man sich, um der steigenden Abonnement-Zahlen Herr zu werden, noch 1978/79 ein EDV-System angeschafft, was in der ‚Szene‘ für Unmut sorgte. Die zuständigen „tazler“ meinten, der Einsatz der EDV wäre, linkes Projekt hin oder her, alternativlos, konterten sogar, dass sie „der Computer von so idiotischen […] Arbeiten wie Adressen erfassen“ entlasten würde; die Informatiker*innen derweil sahen sich im „Mißtrauen gegenüber den Auswirkungen ihrer Wissenschaft unverstanden“ (WILAB 1983: 155).

Auf etwas mehr Verständnis stießen einige der Fühler, die die PROWILA-Truppe bald in andere Richtungen ausstrecken sollte: zu Gewerkschaftlern und Betriebsrätinnen, zu einschlägigen Arbeitskreisen an der TU, zu gleichgesinnten Wissenschaftler*innen im Bundesgebiet. Nebenbei machte man sich schlau: vom Starnberger Max-Planck-Institut aus zirkulierte damals die Vokabel „soziale Naturwissenschaft“; aus den Niederlanden schwappte die Idee „Wetenschapswinkel“ herüber (die *Wechselwirkung* sollte 1979 erstmals davon berichten); aus England und Skandinavien drangen Ansätze „kritischer“ Arbeitswissenschaft, die im Kern um Visionen demokratischer *participation* in punkto mikroelektronischem Fortschritt kreisten (vgl. Stewart-Halevy 2021). Und von Konzepten „alternativer“ Technologie war unter anderem vor Ort zu erfahren, namentlich etwa bei der *Interdisziplinären Projektgruppe für Angepaßte Technologie *(IPAT) – auch im Format (IFP) ein Novum jener Jahre, das neben Sonderforschungsbereich (SFB) und Forschungsprojektschwerpunkt (FPS) der zielorientierteren „Forschungsplanung“ dienen sollte. Genauer handelte es sich, wie ein IPAT-Mitstreiter sich ausdrückte, um einen „fachübergreifenden Forschungsschwerpunkt nach § 50 BerlHG“. „Lässig ausgedrückt sind wir eine Mittelbaugruppe […], durch das unsichtbare Band von Ideen zusammengehalten“ (Schäfer 1984: 11). Zurück ging das unsichtbare Band auf eine Studienreise einiger TU-Studierender nach Indien im Jahr 1969.

Dass derartige, mehr oder weniger konkrete Utopien, offenbar Funktion einer auch „von oben“ begünstigten Nischenbildung, zumindest bei Splittergruppen des FB 20 auf Resonanz stoßen sollten, überrascht nun per se nicht. Im Hintergrund gärten, neben den diversen Krisenerscheinungen des „Industriesystems“, nicht nur die HRG-„Durchsetzung“ und die Verfehlungen der Informatik, denen an der TU schon seit 1971 ein selbstorganisiertes Seminar gewidmet war (Auftaktveranstaltung: „Informatik für Völkermord in Indochina“). Dazu traten Auseinandersetzungen um politisch unliebsame Personalien, „Stellenstreichungen“ sowie latent trübere Zukunftsaussichten – mit 13,5 % etwa lag die hiesige „Akademikerarbeitslosigkeit“ deutlich über dem Bundesdurchschnitt (Schlegelmilch 1982: 401). Und dazu trat das, was Marx-versierte Zeitgenoss*innen als endgültig reelle Subsumtion der Wissenschaft unter das Kapital zu erkennen glaubten. Neuartige Einrichtungen wie der SFB 57 „Produktionstechnik und Automatisierung“ sprachen dafür: „Welche Bedeutung ein derartiges Forschungsprogramm für die Kapitalisten [hat], kann man schon aus den dafür bereitgestellten Mitteln erkennen: 8,174 Mio. DM wurden [bereits] in diesen SFB investiert“ (Zelle Kybernetik 1976: 15).

**Figure 1:**
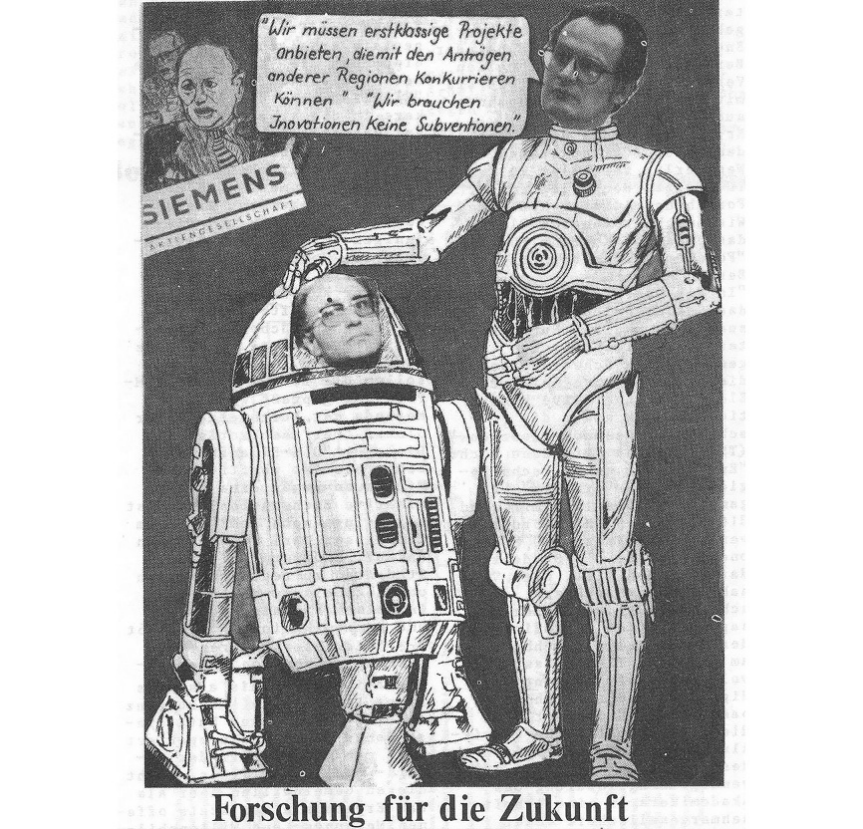

Dass in Zeiten von „Weltmarktkonkurrenz“ und eskalierendem „Verteilungskampf“ sich die Bringschuld der Universitäten massiv potenzierte, war dabei weniger radikale Einsicht, als der Umriss der Diagnose, die auch die neuere Berliner Hochschulpolitik animierte. „Zwang zur Innovation“: das galt für ein („rohstoffarmes“) Land wie die BRD; und das galt umso mehr für ein Quasi-Zonenrandgebiet wie West-Berlin (Allesch et al. 1978: 2). Allein zwischen 1970 bis 1980 schnurrte dort die Zahl der Industriebetriebe von 4072 auf 2381 herunter. Die verbleibenden Betriebe, nicht zuletzt die Großindustrie, so zumal der anschwellende Krisendiagnosediskurs, übten sich bestenfalls in „Scheininnovationen“ – nicht aber im Erfinden zukunftsweisender Produkte, Ideen oder Verfahren. Konzernzentralen und industrielle Forschungslabore hatten sich nach 1945 erst gar nicht mehr in exponierter Lage niedergelassen (vgl. Kreibich 1986: 534ff.; Ahrens 2015). Im Umkehrschluss hieß das: nur (wirkliche) Innovation, nicht „Subvention“, wies den Weg aus der Krise (Abb. 1). „In der Forschung geht es um […] zielgerichtetere Orientierung der Forschungskapazitäten am Problemlösungsbedarf der Gesellschaft“, schrieb der frisch aus Bonn entsandte, notorisch unproletarische Peter Glotz (SPD), seit 1977 Senator für Wissenschaft und Forschung, der einstigen Königlich Technischen Hochschule zu Berlin dann auch in die Festschrift zum hundertsten Geburtstag (Glotz 1979b: X). „Wir müssen den […] Leuten klarmachen, daß die Universitäten Dienstleistungsunternehmen sind“ (Glotz 1979a: 293).

Die „Informatiker wider Willen“ am FB 20 hätten dem in der Analyse wohl sogar beigepflichtet, so wie überhaupt die vielen „Subventionen“ – „Ursache einer deformierten Wirtschaftsstruktur“ – auch in Berliner Alternativ-Kreisen keinen besonders guten Ruf genossen (Nebel-Dampf 1982: 69). Weniger Zustimmung kam den Parametern entgegen, die der versuchten Umorientierung des universitären „Forschungsapparats“ scheinbar zugrunde gelegt wurden. Denn, trotz der nicht wenigen Lippenbekenntnisse in Richtung „sozial gesteuerte Innovation“, mit denen sich die sozialliberale Forschungspolitik der 1970er Jahre dekorierte, lief das Dienstleistungsunternehmen Universität einmal mehr Gefahr, an den eigentlichen Anliegen der Gesellschaft vorbeizuproduzieren.^8^ So meldeten kritische Stimmen dies jedenfalls an. Schon allein, wie die Anti-Festschrift *10 Jahre Fachbereich 20* (1980) festhielt, dass die „Informatik“ beziehungsweise der FB 20 im Wesentlichen ein Produkt der sogenannten DV-Programme des Bundes war, und diese wiederum Reaktion auf Wirtschaftsrezession (1966/67), „technology gap“-Diskurs und IBM-Hegemonie, zeigte, wie sehr das Geschäft mit der Datenverarbeitung immer schon dem Geschäft diente (Beuschel et al. 1980: 8ff.). Ob nun die Programmiersprachen, die an der TU zum Einsatz kamen (wie ALGOL: „Ausdruck der Bestrebungen der europäischen DV-Monopole“) oder die Tatsache, dass einige der TU-Professoren ihre Zeit neuerdings lieber der eigenen Firma widmeten (Beuschel et al. 1980: 35, 127, 174): über die Marschrichtung konnten wenig Zweifel aufkommen.

Öl ins Feuer gossen der Bundesforschungsminister Volker Hauff (SPD, Ex-IBM) und der bereits erwähnte Glotz als diese im Sommer 1978 ihre „Maßnahmen zur Förderung von Forschung und Entwicklung in Berlin“ verkündeten. Um der Malaise entgegenzusteuern, sollten bis 1981 „rund 316 Millionen DM“ gezielt in diverse Schlüsseltechnologie-Sektoren investiert werden (Halbleiter/Kommunikationstechnik, Produktionstechnik, Verkehrs‑, Gesundheits‑, Energieforschung), flankiert von allerlei Begleitoperationen: Investitionszulagen, Personalkostenzuschüsse, eine Berliner „Wagniskapitalgesellschaft“. West-Berlin sollte, wie es überschwänglich hieß, gar zum „Zentrum nachrichtentechnischer Forschung“ ausgebaut werden; ferner wurde die Einrichtung eines städtischen „Technologietransfer-Verbunds“ beschlossen (Burmeister & Canzler 1988: 33; Anonym 1978b: 2).

Es war an der Zeit, die „Isolierung der Hochschulen [zu] durchbrechen“, so Glotz. „Mich beunruhigt keine Sekunde die Verdächtigung“, notierte er trotzig ins (umgehend publizierte) Tagebuch, „wir täten dies alles nur für die ‚langfristige Absicherung des kapitalistischen Verwertungsprozesses‘“ (Glotz 1979a: 164). Der damit einhergehende und zumindest verbal eingeforderte „Abbau esoterischen (konsequenzlosen) Theoretisierens“, der (tatsächlich implementierte) „Sparkurs“, die steigenden Dritt- und Projektmittel, der Abbau von „dysfunktionale[n] Formen der Mitbestimmung“ (Glotz 1980: 92, 94; Niederwemmer et al. 1978: 234) – all das verlief freilich alles andere als reibungslos, trotz der vielen Beteuerungen, dass die universitäre Forschung weiterhin „unabhängig und unbeeinflußt“ vonstattengehen würde und die Berliner Politik sich jedenfalls an höheren Zielen orientiere. (So legte Glotz 1980 nach: „10 Thesen zu einer sozialorientierten Forschungspolitik in Berlin“). Wie *Die Zeit *damals zu wissen glaubte, galt es an Berliner Universitäten zwar ohnehin „als unfein, für die Bedürfnisse der ‚kapitalistischen Wirtschaft‘ zu forschen“ (Nawrocki 1978); das Potenzial aber, so waren sich Beobachter einig, war da – und wuchs: schließlich war der Öffentliche Dienst zwischenzeitlich der einzige „Sektor“, der in West-Berlin noch expandierte beziehungsweise zwecks Kompensation „aufgebläht“ wurde.^9^

Einig war man sich dort in Sachen Zukunftsfragen nicht. Der damalige TU-Präsident und (wie man munkelte) „verlängerter Arm“ Glotz’, Rolf Berger, verlor über solche Richtungskämpfe prompt sein Amt (Anonym 1978c). Relativ unbeirrt trug dessen (derart unkündbare) Nachfolger Starnick die „Hightech-orientierte“ Strukturpolitik des Senats weiter, was nicht nur im Umfeld des PROWILA skeptisch beäugt wurde. So etwa: der 1978 aufgegleiste Partnerschaftsvertrag mit dem Massachusetts Institute of Technology (MIT: die „größte Kriegsforschungsuni in den USA“) (Anonym 1983b: 28); Kooperationsverträge unter anderem mit Siemens, IBM, Schering; das Kabelpilotprojekt BERKOM; Erweiterungen des Heinrich-Hertz-Instituts; der Elektronenspeicherring „Bessy“ (Halbleiter); und das Fraunhofer-Institut für Produktionsanlagen und Konstruktionstechnik (IPK), das in Personalunion mit dem erwähnten SFB 57 geführt wurde. 1982, nun schon im raueren Wind des „Wende“-Klimas, besetzten „etwa 60“ Soziologie-Studierende der FU eben jenes Fraunhofer-Institut mit der Begründung: „weil es von den bei uns weggekürzten Geldern finanziert wird; weil die dort betriebene Forschung […] im Interesse der Rationalisierung der Produktion betrieben wird“. Im Gegenzug forderten die Besetzer*innen „selbstbestimmte Forschung […] ökologisch angepaßte Produktion, angepaßte Technologie“ (Anonym 1983b: 25–26).

Vergeblich. Vorrang hatte die „Modernisierung der Volkswirtschaft“ (Scharpf & Hauff 1975). „Es geht nicht darum, Berlin zu einer Modellstadt zu machen“, gab der nicht nur lokal einflussreiche Innovationstheoretiker Gerhard Mensch (WZB) zu Protokoll, „sondern darum, ein paar Basisinnovationen, die ohnehin anstehen, statt sie anderswo geschehen zu lassen, nach Berlin zu holen“ (Mensch 1979: 100; vgl. auch Weber 2018: 95ff.). Die Unausweichlichkeit der Entwicklungen stand den Kondratieff-Zyklen, mit denen Mensch gerne hantierte, ins Gesicht geschrieben.

## „Unternehmergeist in traditionsreicher Umgebung“

Eine mittlere Flut an Expertisen zur Animation des hiesigen Innovationsklimas wurde infolge verfertigt, unter anderem am erwähnten Wissenschaftszentrum Berlin (WZB), von wo aus bereits die programmatische Rede von der „Modernisierung der Volkswirtschaft“ in Umlauf gebracht worden war.^10^ Zudem steuerten bei: das 1972 lancierte Fraunhofer-Institut für System- und Innovationsforschung (ISI) in Karlsruhe; Günter Spur (der Direktor des verhassten Fraunhofer IPK); und Rolf Kreibich, der ehemalige FU-Präsident und Zukunftsforscher.^11^ Expertengruppen tagten; Studienreisen wurden durchgeführt (Route 128, Cambridge Science Park usw.); der Chef der Berliner IHK forderte in seiner Neujahrsansprache 1979 im Fernsehen eine „Innovationsoffensive“.

Das alles barg offenbar Konfliktpotenzial – nicht alle etwa teilten Wissenschaftssenator Glotz’ Auffassung, dass der Staat „direkt Forschung veranlassen“ solle und dürfe (Glotz 1980: 94). (Schon eher die, dass nicht mehr einfach mit der „Gießkanne“ gefördert werden könne). Glotz selbst verzweifelte denn auch nicht nur an den „patentierten Marxisten“ sondern gleichermaßen an der Industrie, die, apropos Sozialverträglichkeit, über „‚F- und E‑Personalkostenzuschüsse‘ […] fast so viele Putzfrauen abschr[ieb] wie Wissenschaftler“ (Glotz 1980: 94–95; 1984: 130).

Und, *more to the point*: der innovationspolitische Aktionismus zeitigte – keineswegs eineindeutige – Effekte auch in jenem Milieu, das dem Treiben aus kritisch-sozialbewegter Warte allermindestens skeptisch gegenüberstand. Die neuen „Kräfteverhältnisse“, die sich nun latent zugunsten von Professoren und Präsidenten verschoben, wie auch der krisenbedingt eingeschlagene „Sparkurs“ führten dazu, dass man sich dort zusehends mit dem Rücken zur Wand befand – oder sich jedenfalls so fühlte. Das Ingenieurskollektiv Wuseltronick, das sich um 1977 von der TU abnabelte, etwa wollte lieber „selbst entscheiden, was mit unserer Forschung geschieht“ und weg von der „perversen Verwendung von Forschungsergebnissen“ (Christ 1981). Auch die ursprüngliche Idee, den WILAB wie die Vorbilder aus den Niederlanden* innerhalb* der TU zu verankern, war, so musste man sich spätestens 1981 eingestehen, „im ‚Zeitalter‘ der Mitteleinsparungen […] in weite Ferne gerückt“ (Bickenbach & Keil 1981: 44). Umgekehrt produzierte der große Zukunfts-Push, wie bereits angedeutet, durchaus neue Nischen. Die intensivierten Beziehungen zur „Kriegsuni“ MIT etwa dürfte mit ein Grund gewesen sein, dass im Wintersemester 1979/80 der Computerkritiker-vom-Dienst, Joseph Weizenbaum, am FB 20 als Gastprofessor in Erscheinung treten konnte (Beuschel et al. 1980: 143). Und schon 1978 rekrutierte die TU die Informatikerin Christiane Floyd. Dies in der Hoffnung, sie möge dort „zwischen den Extremen schlichten“ – im Dunstkreis der unkonventionellen Professorin, die „1968“ in Kalifornien verbracht, und sich vom „verkopften“ Rationalismus der Informatik also bereits ein Stück weit entfremdet hatte, versammelte sich dann auch das Personal des WILAB (Siefkes et al. 1999: 137). Ferner wäre da: das (kontroverse) BMFT-Programm „Humanisierung des Arbeitslebens“ (1974–1989), das ebenfalls in den Sog der „neuen“ Technologien geriet (Kleinöder et al. 2019). Von letzterem profitierten vor Ort nicht nur Siemens und die „bürgerlichen Apologeten“ des SFB 57 (Anonym 1977b), sondern etwa auch der unbequeme TU-Psychologe Walter Volpert. Volperts zunehmend vehement vorgetragene Plädoyers für eine „neue Arbeitswissenschaft“, eine „Aktionsforschung“ im „Interesse der Arbeitenden“ (Volpert 1978: 115), sollten, wenig überraschend, auch im WILAB-Dunstkreis einigen Eindruck machen. Es sollte nicht lange dauern, bis sich derartige Wege kreuzten.

Innerhalb des „Forschungsapparats“ konnte offenbar dennoch schnell der Eindruck entstehen, dass sich die Zeit der Experimente dem Ende neigte. Ganz anders jenseits des Elfenbeinturms: die Berliner *Alternative Liste* (AL) wurde im Herbst 1978 ins Leben gerufen, in der *Neuen Welt*, nur ein paar Gehminuten vom späteren WILAB entfernt. Geschätzte 100.000 Besucher*innen strömten im selben Jahr zum *Alternativen Umwelt-Festival Berlin* (Uhde 1979). Im nahegelegenen Wendland brauten sich „atomare Gefahren […] für die Insel Berlin“ zusammen – Gorleben (Anonym 1978a: 427). Und, legendär, Ende Januar 1978 im Audimax der TU: der TUNIX Kongress (Falasca et al. 2018), bei dem Glotz versuchte (wofür er besser in Erinnerung geblieben ist), den zahlreich anwesenden Vertreter*innen der „Sponti-Bewegung“ Vernunft einzureden: „Die herrschende Klasse, glauben sie, hat alles geplant“ (Glotz 1979a: 257). 1981 zog das AL ins Rathaus ein, noch vor der FDP. Und nicht minder einschneidend, quasi als vorgezogene „geistig-moralische Wende“: fortan regierte in West-Berlin die CDU. Für „bundesweit[e] Furore“ sorgte infolge nicht zuletzt der frisch gekürte Sozialsenator Fink beziehungsweise dessen mit „Staatsknete“ versüßten Umarmungstaktiken der Szene – der Graben zwischen „Konservativen“ und „Alternativen“ wäre in mancherlei Hinsicht nämlich gar nicht so groß, so Finks Einsatz, verbindende Elemente fänden sich in Sachen „Hilfe zur Selbsthilfe“ und „Entstaatlichung“ (vgl. Fink 1983; Mayer 1987).

PROWILA beziehungsweise der WILAB, der 1981/82 ebenfalls konkrete Formen annahm, sollte (wie viele andere Projekte auch) trotz zeitweiliger „Konzentration der Kräfte“ auf das Dickicht von Vergabekriterien und Antragsverfahren von solcher „Staatsknete“ nichts sehen (Beuschel et al. 1984: 36, 38). Wie Beobachter*innen der lokalen Politszene schnell bemerkten, änderte sich in manchen Belangen gar nicht so viel. Befreit von „sozialliberalem“ Beiwerk, forcierten die (fast ausnahmslos) neuen Stadtväter insbesondere die bereits eingeschlagenen Wege der „Innovationspolitik“. So pries „der Senat bei jeder Gelegenheit die Wissenschaftsszenerie“ der Inselstadt: 100.000 Studierende, 12.000 Wissenschaftler*innen, 200 Forschungsinstitutionen (Burmeister & Canzler 1988: 61). Der Wirtschaftssenator, Elmar Pieroth, wollte mit den „Japanern“ gleichziehen (Anonym 1982b). Der Wissenschaftssenator Prof. Wilhelm Kewenig gab zu Protokoll, schon einmal mit eigenen Augen gesehen zu haben (als Student in Harvard), wie sich eine darniederliegende Stadt – Boston – via Hightech aus dem Sumpf gezogen hatte (Staatsministerium Baden-Württemberg 1984: 173–174). „West-Berlin Reaches for Old Glory in Science“ (Sullivan 1988), so bemerkte man selbst im Ausland, was auch daran gelegen haben mag, dass die technowissenschaftlichen Aufbrüche von ehedem nun verstärkt auch ins Visier der Historiker*innen gerieten. Sei’s in kritischer oder monumentaler Absicht: an einschlägigen Ausstellungen, Industriewanderpfaden, Stadtteilprojekten, überhaupt an Jubiläen herrschte damals kein Mangel.^12^

**Figure 2:**
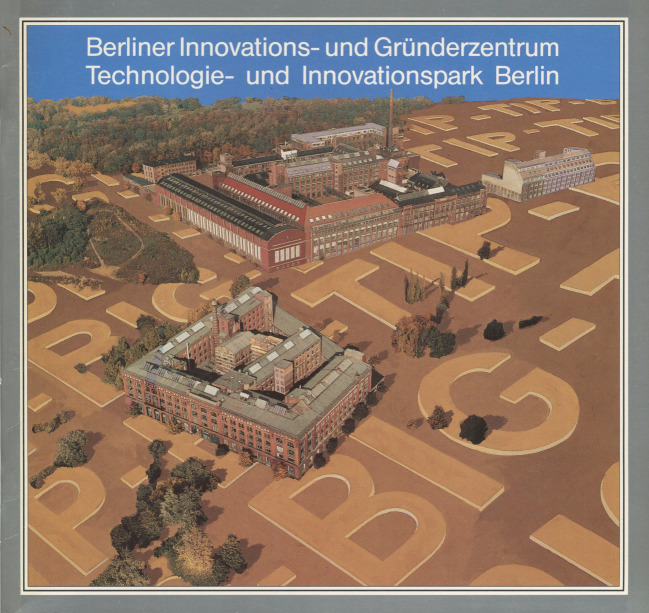

Als Herzstück derartiger Standortpropaganda fungierte der bereits erwähnte, von TU und Senat angeschobene „Wissenschaftsladen von Kapitalisten für Kapitalisten“: das im November 1983 feierlich eingeweihte *Innovations- und Gründerzentrum* (BIG), das erste derartige Zentrum auf deutschem Boden überhaupt (Abb. 2). „Es gab schon einmal Gründerjahre“, versprachen die BIG-Broschüren, die ausgiebig damit kokettierten, dass sich dies alles – „Silicon Wedding“ – auf unlängst ausrangiertem AEG-Gelände zutrug (BIG 1985: 1). Noch 1976/77 hätte man dort wütende Plakate vorfinden können: „Leere Räume bräuchten nicht [zu] sein – bringt man Arbeit nach Berlin herein“;^13^ bis 1985 wären dann immerhin bereits „über 4000 Besucher durch das Zentrum geschleust worden“, notierte die *Wechselwirkung*. Die davon ausgehende ideologische „Katalysatorfunktion“ sei allerdings nicht zu unterschätzen: schließlich passe das „propagandistische Lieblingskind“ der neueren Forschungs- beziehungsweise Stadtpolitik, dem im BIG ein ideales Zuhause bereitet werden sollte, nur zu gut zur geistigen Situation der Zeit (Schlag 1985: 47–48). Die Organisator*innen einer „anti-militärischen Stadtrundfahrt“, die eher assoziativ auch am BIG vorbeiführte, stellten diesbezüglich heraus, dass hier „Jungdynamische Jungunternehmer […] mit Steuergeldern neue Technologien bis zur Marktreife [entwickeln]“, unter Rückgriff auf Infrastrukturen der TU, von Risiko und Unternehmertum also eigentlich keine Rede sein konnte (Anonym 1985a, 107–108).^14^

Dass noch im Juni 1984 ein Brandanschlag aufs BIG verübt wurde, fügte sich durchaus ins Bild. „BIG steht für Fortschritt“, stand im Bekennerschreiben, „aber nicht den, den wir wollen“ (Anonym 1985a: 108). Vom BIG, überhaupt von „Technologieparks“ – BRD-weit zählte man 1986 bereits 41 solche Einrichtungen –, waren dennoch viele angetan. „Venture Factory: West-Berlin fuses corporate and academic life“, titelte die *Financial Times*, „no sign of ‚Eurosclerosis‘ there!“ (Carr 1985). Angetan: heißt, von der dem BIG zugrunde liegenden Idee, „Gründern bei ihrer Arbeit“ zu helfen, wie eben etwa durch die Bereitstellung von Infrastruktur (Kopierservice, Getränkeservice, Konferenzräume usw.) (BIG 1985: 6–7); von universitären Ausgründungen, die am BIG Unterschlupf fanden, wie *Trion Präzisionselektronik* oder *Ro-Ber Industrieroboter GmbH*; und überhaupt vom neueren Berliner Unternehmergeist, der hier und dort also trotz allem aufblitzte und womöglich vom Ende der „anti-entrepreneurial influences“ an deutschen Universitäten kündete (Aspen Institute 1985: 6).

Nicht zuletzt hatten sich einige Zeitgenoss*innen offenbar von der elementaren Wichtigkeit von „Transfer“ überzeugt: dies nicht nur aufgrund der zunehmenden „Notwendigkeit für die Hochschulen, mehr finanzielle Mittel [= Drittmittel] zu erschließen“, was einen Rattenschwanz an hochschulpolitischen „Flexibilisierungen“ nach sich zog (Nebentätigkeitsregelungen, Zeitverträge usw.) (Allesch & Preiss-Allesch 1986: 271). Der Mangel an Transfer – von Wissen, Technologie, Personal – zwischen Universität und Unternehmen, insbesondere kleiner und mittelständischer Unternehmen – blockiere, wie es schon damals hieß, „Innovation“ und damit Wege aus der „Depression“ (Mensch 1975).^15^

**Figure 3:**
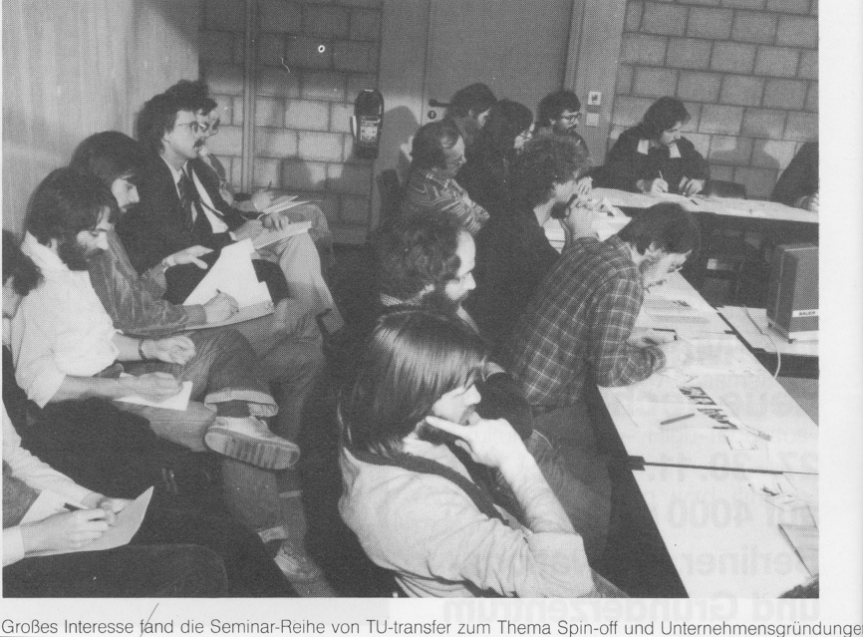

Im Hintergrund agierte (auch wenn die örtliche CDU sich das BIG nun gern selbst auf die Fahnen schrieb) konsequenterweise die 1977/78 ins Leben gerufene Transferstelle, die nicht nur das Gründerzentrum, sondern auch flankierende Neuerungen wie den „Forschungsmarkt Berlin“, „Spin-off“-Seminare und die lokale Gründermesse *BIG-TECH* anschob (Abb. 3). Gemäß dem Leiter von *TU-transfer*, Jürgen Allesch, befand sich die TU damit an vorderster Front in punkto „Öffnung der Hochschule hin zur Gesellschaft“ (Allesch & Krug 1982: 4). In Zeiten von „Marktsättigung“ und intensivierter „Konkurrenz auf dem Weltmärkten“ standen mit dem Typus des „früher viel verlachten ‚Bastler[s]‘, ‚Tüftler[s]‘ und unkonventionellen Ingenieurerfinder[s]“, so Allesch, gar „neue Gründerjahre“ ins Haus (Allesch 1984: 407): Rund 400 „konkrete Kooperationsprojekte zwischen Hochschule und Praxis“ waren bis 1983 durch *TU-transfer* angeleiert worden, darunter die Entwicklung eines Gehörschutzes („Radaunix“), eines Software-Entwicklungssystems („CDL-Labor“) und eine Unternehmung im Segment „Wassersport und Meerestechnik“ (Aquata).

## Kreuzberger Luft

Mit solch universitärer Umtriebigkeit, die Ende der 1970er Jahre noch mit einem durchaus „sozial-orientierten“ Anstrich daherkam, sich von solcher Rhetorik aber zunehmend entfernte, musste man sich also messen: sei es weil, wie der Wuseltronicker Reiner Lemoine befand, stets die Gefahr bestand, damit letztendlich „voll in den Rahmen der CDU-Politik [zu] fallen, […] halt universitäre Forschung […] in Produkte umsetzen und dann verkaufen“ (Bolda et al. 1988: 88). Oder sei es weil, wie man am WILAB glaubte, die eigentlich „Betroffenen“ im Kundenkreis von *TU-transfer* und ähnlichen Vorstößen gar nicht erst vorgesehen waren.^16^ „Greift man den Anspruch der Hochschulen, Servicezentrum für alle gesellschaftlichen Bereiche zu sein, auf, dann bleibt bisher ein großes Defizit […] übrig“, wie Joachim Bickenbach, einer der WILAB-Gründer*innen, dies 1983 nach einem knappen Jahr Ladenarbeit bilanzierte. Die dem Wissenschaftsladen zugedachte Rolle war so gesehen die, eine *andere*, „neue Art von Transferstelle“ zu bilden: eine, deren „Zielgruppe“ nicht nur aus mittelständischen Unternehmen oder, schlimmer noch, aus der Großindustrie bestünde (Bickenbach 1983: 287–288). Letztendlich müsse es also um das gehen, um was es am BIG kaum gehen konnte: nämlich darum, den „Mangel an demokratischer Legitimation bei der Ausrichtung des ‚wiss.-technischen Fortschritts‘“ zu beheben (Diekmann 1986: 422).

Konkreter hieß das: „gesellschaftlichen Gruppen Zugang zur Wissenschaft [zu] verschaffen […], den diese aus den verschiedensten Gründen kaum oder gar nicht haben“ (Diekmann 1986: 421). Auch hinsichtlich dieser Transfer-Missstände bestand ein gewisser Bedarf: bis 1985 war der WILAB „über 650 mal in Anspruch genommen“ worden (Diekmann 1986: 415). Zu diesem Zeitpunkt, gewissermaßen am Peak auch der „WILA-Bewegung“ – bundesweit zählte man 1985 ca. 65 „alternative“ Wissenschaftseinrichtungen (Brockner 1985: 10) – war die Ladenbesatzung von den ursprünglichen fünf Informatiker*innen auf knapp 60 Mitglieder angewachsen. 15 davon bildeten den „harten Kern“, die sich im rotierenden Ladendienst abwechselten; zeitweise konnte man sogar eine „Ladenstelle“ finanzieren (wenn auch eine mit „Mini-Lohn“). Der Anfragen jedenfalls konnte man sich zunächst kaum erwehren: knapp über die Hälfte (Stand Dezember 1983) betraf die „Neuen Technologien“, gefolgt von den Themenkomplexen Umwelt/Ökologie, Leben/Wohnen/Soziales und „Sonstiges“, so etwa die beratende Unterstützung einer Person, die „einen ‚alternativen‘ Kühlschrank für Entwicklungsländer konstruiert und […] auf Kreta bereits ausprobiert“ hatte (Beuschel et al. 1984: 37; Anonym 1982a: 53). „Der zur Bearbeitung der eintreffenden Anfragen notwendige Aufwand“, soviel war schnell klar, „übersteigt die verfügbare Zeit der ehrenamtlich tätigen Ladenmitglieder“ (Diekmann 1986: 415).

**Figure 4:**
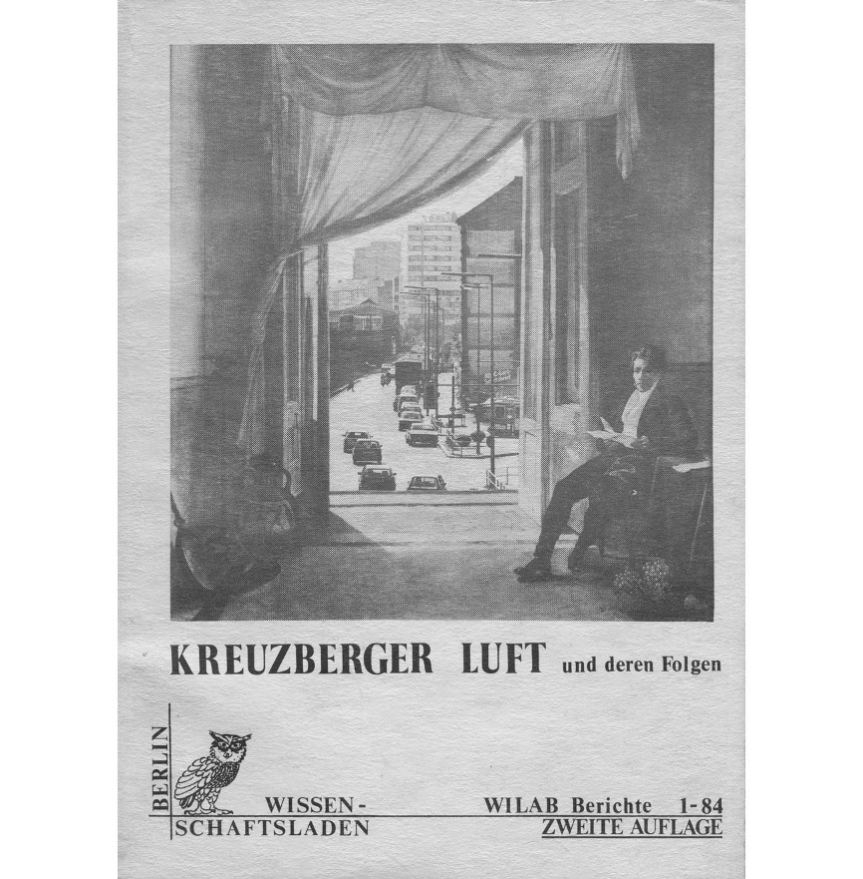

Drei Arbeitsschwerpunkte sollten sich dennoch herausschälen, die auch die bald schon sich diversifizierende Expertise der Ladenbetreiber*innen reflektierte: Erstens, das „Smog-Projekt“, das von Bürgerinitiativen noch 1982 an den WILAB herangetragen worden war. Resultat war die AG Luft, die sich der notorischen Luftverschmutzung Berlins verschrieb (Abb. 4). An mehreren Kreuzberger Standorten wurden im Rahmen der AG-Aktivitäten Schwefeldioxid-Messungen durchgeführt – als „autonomes“ Gegenprogramm zu den Messreihen des Berliner Senats (Gass et al. 1984: VII). Zudem bot sich hier die Möglichkeit, die neueren Informationstechnologien einem demonstrativ guten Zweck zuzuführen. Den vielen Messdaten – insbesondere den ebenfalls verfügbaren („offiziellen“) Senats-Daten, die im 3‑Minuten-Takt von 31 Messstationen hergestellt wurden – war ohne Rückgriff auf die EDV kaum Herr zu werden (Beuschel et al. 1983b: 5). Zweitens, der Arbeitsbereich „ABM-Beratung“, in Kollaboration mit *Netzwerk Selbsthilfe e.* *V.* und dem im Zuge der Fink’schen Charmeoffensive lancierten „Arbeitskreis Staatsknete“: Bedarf bestand hier aufgrund der zunehmenden „finanzielle[n] Austrocknung“ vieler Projekte und der undurchsichtigen Vergabekriterien (Beuschel et al. 1984: 36). Und schließlich, als „Dauerbrenner“: der Arbeitsbereich „Informationstechnologien am Arbeitsplatz“, wobei das angebotene Dienstleistungsspektrum dem der Transferstelle *TU-transfer* hier tatsächlich durchaus ähnelte – auch letztere verstand sich in erster Linie als Informations- und Vermittlungsagentur, dazu auserkoren, die vielfältigen „Hemmnisse“ abzubauen, die der engeren Liaison von Theorie und Praxis noch im Wege standen (vgl. Allesch 1981). Ähnlich am WILAB, freilich unter deutlich anderen Vorzeichen und Bedingungen: Anfertigen von (alternativen) Gutachten, Vermittlung von Sachverständigen, Beschaffung von wissenschaftlicher Literatur, Übertragung derselben in „allgemeinverständliche Form“, Diskussionsabende durchführen – all das gehörte mit zum Portfolio (WILAB 1983: 172).

„Leitlinie dabei [war]: Hilfe zur Selbsthilfe“ (Beuschel et al. 1983b: 3). Heißen konnte das beispielsweise: „Verhinderungsstrategien“ für die oder besser: mit den zumeist un- und/oder desinformierten Betroffenen zu entwickeln – etwa in Hinblick auf „Personalinformationssysteme“. Nicht auszuschließen, war diesbezüglich in der *Wechselwirkung *zu lesen, dass derartige Systeme, die bei Siemens, Daimler und AEG bereits ausgerollt wurden, „Verhaltensdaten“ irgendwann gleich mit erfassen würden (Beuschel et al. 1984: 35; Degenhardt 1980: 17). Oder, und konstruktiver, es ging darum, „Gestaltungsalternativen“ aufzuzeigen und wenn möglich auch durchzusetzen, was prinzipiell immer und überall der Fall war, wo EDV-Experten am Werk waren beziehungsweise wo „Bildschirmarbeitsplätze“ eingeführt werden sollten. Das wiederkehrende Problem dabei: nicht nur geschah das in aller Regel über die Köpfe der betroffenen Menschen hinweg, es wurde dabei eine „leblose Struktur […] den lebendigen Prozessen […] aufgeprägt“ (Floyd 1987: 72).

Die lokale Gegenexpertise des WILAB, die im Gegenzug nun konkretere Formen annahm – sei’s in Interaktion mit widerwillig computerisierenden Alternativprojekten (vor allem im „Druck- und Bio-Food-Bereich“), sei’s durch Feldkontakt mit Betroffenen in „bürgerlichen Betrieben“ (WILAB 1983: 29, 126) –, haderte folglich mit einer ganzen Reihe an Dingen. Anstoß war etwa daran zu nehmen, dass EDV-Systeme bei Einführung dazu neigten, Arbeitsabläufe nach den Vorgaben der Software umzustrukturieren, nicht umgekehrt, und dabei bestehende, „sinnvolle Arbeitszusammenhänge […] zerstückelt“ wurden (Diekmann 1986: 416). Ferner zogen real existierende EDV-Systeme deshalb tendenziell Formen der Entqualifizierung nach sich. Und nicht zuletzt an der dem allen zugrunde liegenden Ideologie konnte man verzweifeln: an der suggerierten Alternativlosigkeit dieser Entwicklungen einerseits, und am reduktionistischen Menschenbild der Informatik anderseits. „[D]ehumanizing“ wäre die (spontane) Psychologie derjenigen Fachvertreter, denen nur daran gelegen war, „user behavior“ arbiträren Systemlogiken zu unterwerfen, konstatierten Floyd und der künftige WILAB-Gründer Keil 1981 – hier in ihrer Kapazität als Professorin beziehungsweise als Assistent am FB 20 (Floyd et al. 1981: 491). Der erwähnte Arbeitswissenschaftler Volpert, mit dem Floyd und Keil 1981/82 im Rahmen des BMFT-geförderten Projekts ISAR („Informationssystem Analyse und Restrukturierung“) erstmals Kontakt aufnahmen, sah bereits einen „neuen Taylorismus“ heraufziehen (vgl. Floyd 2002: 436).

Solche Überzeugungen verschärften sich mit zunehmender Versenkung in die Wirklichkeiten des EDV-Alltags oder, wie es am WILAB geheißen hätte, im Vollzug betroffenenorientierter Wissenschaft. „[It] enabled us to learn a great deal about […] their everyday [computer] use“, so sollte Keil dies rückblickend festhalten. Und: es provozierte die notwendigen Blickverschiebungen – „from the developer’s to the user’s point of view“ (Keil-Slawik 1987: 9).^17^ Anschauungsmaterial dazu gab es vor Ort zur Genüge: insofern die mikroelektronische Rationalisierungswelle schon längst auch die Büros erreicht hatte, also nicht zuletzt den so geschmähten, weil „aufgeblähten“ Öffentlichen Dienst. Und insofern die „Deindustrialisierung“, wie anderswo auch, nicht nur leere Fabrikhallen und attraktivere Kulturangebote mit sich brachte, sondern auch Arbeitsintensivierung und neue Formen der Fremd- und Unterbeschäftigung (die florierende Alternativökonomie wäre selbst ein Beispiel). Dass sich 1977 die neue, Dynamik-ausstrahlende Berliner SPD/FDP-Regierung anschickte, testhalber „Stechuhren“ bei den Behörden einzuführen, konnte man da noch als ominöses Kuriosum verbuchen (Anonym 1977a); durch den „Einsatz von Schreibautomaten, neuen Telekommunikations- und Informationssystemen“, errechnete der DGB Landesbezirk Berlin nur wenige Jahre später, fielen im Zuge der „Rationalisierungsstrategie“ des Senats „im Durchschnitt zwei von drei Arbeitsplätzen fort. Mittlerweile sind ¾ aller Schreibarbeiten zentralisiert. Zunehmend werden Sachbearbeiter mit diesen ‚Nebentätigkeiten‘ betraut“ (DGB Landesbezirk Berlin 1984: 83).

## Kritik und Krise

Die Anliegen des Projekts WILAB, angesiedelt irgendwo zwischen informierter „Verhinderung“ und „menschengerechter Gestaltung“ jener damals neuen Technologien, trafen sich dann auch mit dem, was sich hier und da, und für eine Weile, nicht nur in West-Berlin oder dem übrigen Bundesgebiet als Hightech-affines „Gegenwissen“ artikulierte. Auch und insbesondere in Schweden, Norwegen und Großbritannien formierten sich, freilich tendenziell in Form von Rückzugsgefechten, damals die Gegenkräfte. Dank Kanälen wie *Wechselwirkung* konnte man auch in West-Berlin davon allermindestens lesen: Projekte mit vielsagenden Namen wie UTOPIA und DEMOS; kommunale Experimente wie das Greater London Enterprise Board (GLEB); schillernde Begriffe wie „democratized planning“, „socially useful production“, „human centred systems“ (z. B. Cooley 1979; Hinderhofer & Deuring 1980). Die Konsequenzen solcher Initiativen blieben bekanntlich überschaubar – in Zeiten eskalierender Forschungs- und Entwicklungskosten, „internationaler Arbeitsteilung“ und angeschlagener Gewerkschaften, gerann die bald schon geflügelte Rede von der *social construction of technology* zum vorwiegend theoretisch-akademischen Unterfangen.

Was den Kreuzberger Wissenschaftsladen betrifft, verband sich damit (allerwenigstens) die Hoffnung, nicht mehr nur „Fachidiotie“ (wie am FB 20) zu betreiben, und dies unter Bedingungen – der Eindruck konnte zwischen „neokonservativen“ Gegenreformen und Hightech-Fieber offenbar leicht entstehen –, unter denen das immer weniger vorgesehen war. Und, sicherlich auch dies ein Grund des Scheiterns: fast notwendigerweise begab man sich damit an die Grenzen der jeweiligen Disziplin. Die „Anfragen an den Wissenschaftsladen“, hieß es 1986 schon nicht mehr ganz so zuversichtlich, „sind dadurch gekennzeichnet, daß sich wechselseitig beeinflussende soziale, ökonomische, organisatorische, technische und rechtliche Fragestellungen auftauchen, die zudem durch innerbetriebliche Konfliktpotentiale überlagert werden“ (Diekmann 1986: 416).

Ein merklicher Effekt derartiger Überlagerungen war etwa der, dass auch den Diskurs des WILAB schnell ein anderes, ökologisch-systemisches Vokabular zu durchziehen begann.

Die Abkehr vom strammeren Diskurs der „Roten Zelle Kybernetik“, die noch in den 1970er Jahren im Einzugsbereich des FB 20 den kritischen Ton angab (Beuschel et al. 1980: 32–33), war dabei teilweise in der Informatik angelegt („systems“ u. Ä.) (vgl. Siefkes 1983); teilweise Nebenprodukt der pragmatischeren Vision „Betroffenen-Beteiligung“; und teilweise Effekt des neueren holistischen Zeitgeists – von Foerster, Bates, Maturana/Varela –, der auch bald schon am FB 20 kursierte (z. B. Keil-Slawik 1985). Prinzipiell aber war dieses neue Vokabular ein Vehikel, den Anspruch, als Ingenieurswissenschaftler*in reflektierter zu agieren, auch irgendwie einzulösen. Über die „Umwelt der Informatik“, so hatten sich die WILAB-Macher*innen ja schon lange überzeugt, lernte man im Elfenbeinturm herzlich wenig – und diese Umwelt war *komplex* (Keil 1983: 165, 167).

Noch deutlicher ins Auge aber fallen die engen Grenzen, die solchen Zielsetzungen beziehungsweise – der Vorwurf ließ nicht lange auf sich warten – Träumereien größtenteils gezogen waren (so z. B. Tontsch 1987). Quasi-Szene-typisch herrschte am WILAB chronischer Ressourcenmangel. Bauen konnte man, wie skizziert, in solchen etwas zu engagierten Fällen weder auf „Staatsknete“ noch auf die Unterstützung von „finanziell (gut) abgesicherten Sympathisanten (Professoren zum Beispiel)“ (Beuschel et al. 1984: 38). (Ein von der AG SPAK organisiertes Treffen 1984 in Göttingen – „Finanzierung und Professionalisierung von Wissenschaftsläden“ – hatte etwa genau diese Problematik zum Thema.) In jedem Fall mangelte es an Kapazitäten: Was sollte man, sinnierte ein Vertreter des WILA Köln, mit 1000 DM im Monat schon ausrichten gegen den „herrschenden Wissenschaftssektor mit seinem zig-Milliarden-Etat“? (Brockner 1985: 13). Und „[a]ußerdem [wäre] immer zu fragen“, inwieweit so ein selbstverwalteter Laden, wenn nicht gleich der allseits betriebenen „Privatisierung der Forschung“, so doch mindestens der Absonderung „kritischen Potentials“ aus den Universitäten zuarbeite (Schäfer 1985: 45–46).

In gewisser Weise ähnelten derartige Probleme damit jenen, die auch jedes „Spin-off“ geplagt hätten – „Streß, viel Improvisation und Frust ist für viele der 33 Jungunternehmer im BIG die Bilanz der ersten Gründerjahre“, so die *Berliner Morgenpost* 1986 (Winkler 1986: 1). Dies trotz der Bemühungen der Berliner Wagniskapitalgesellschaft; dies trotz der Bemühungen um mehr „Beweglichkeit in der [universitären] Personalpolitik“; und auch trotz der vielen Bemühungen (Gründerseminare, „Forschungsmarkt“ u. Ä.) von *TU-transfer*, die Universitätsinsassen überhaupt zum Gründen zu bewegen (Allesch & Preiss-Allesch 1984: 14–15). Im Zweifelsfall ließ sich mit der Aussicht auf Profit dennoch einiges ausrichten. Man nehme: das BIG-Vorzeigeunternehmen *Ro-ber Industrieroboter GmbH*, eines der knapp 15 Unternehmen, die bis Mitte der 1980er Jahre im Dunstkreis des SFB 57/Fraunhofer IPK entstanden waren. Apropos „Startbedingungen“ konnte man hier 1984 immerhin auf 900.000 DM Fördermittel des BMFT verweisen, zudem 1.000.000 DM vom Berliner Innovationsfonds, 1.000.000 DM Darlehen vom Land Berlin sowie 1.500.000 DM an Bankkrediten (Schlimm 1984: 132). Im Fall WILAB – apropos Konsequenzen – diffundierten einige Aspekte deren Feierabendtätigkeit immerhin zurück in den Elfenbeinturm. So trug etwa das, was zeitweilig als „Berliner Konzept des Software-Engineering“ eine gewisse Sichtbarkeit auch in Fachkreisen genoss, deutliche Spuren des Engagements jenseits des FB 20. Namentlich unter anderem STEPS: „Softwaretechnik für evolutionäre, partizipative Systementwicklung“, ein methodischer Ansatz, der versprach, ganzheitlicher, prozess-orientierter, auch empirischer zu sein; es ging – wie die Informatikerin Fanny-Michaela Reisin, die ebenfalls im Floyd/WILAB Umfeld ihr Handwerk lernte, sich ausdrückte – oder sollte gehen: um Software-Engineering als „emanzipatorische Herausforderung in Richtung Wirtschaftsdemokratie“. Dies wäre nicht zuletzt auch ein „weibliches Anliegen“ (Reisin 1992: 242).

Auch das blieb, wie man sich unschwer vorstellen kann, größtenteils frommer Wunsch. Von einigen Ausnahmen abgesehen – das 1984 aufgelegte NRW-Landesprogramm „Mensch und Technik“ etwa förderte ein entsprechendes Projekt – kamen derartige Ansätze kaum vom Fleck. Industrielle „Kooperationspartner“ fanden sich keine; Kooperation wäre, so Floyd, auch gar nicht erwünscht: bestenfalls ginge es der Industrie um „Pipifax-Ergonomie“ (Floyd 1987: 73).^18^ Dass inner- wie außerhalb der Elfenbeintürme ein noch kälterer Wind zu wehen begonnen hatte, half nicht weiter: eine neue Dimension „neokonservativer Wissenschaftspolitik“ war in den Augen dissidenter West-Berliner Wissenschaftler*innen erreicht, als sich Senator Kewenig 1985/86 anschickte, mit der Reinstallation einer („elitären“) *Akademie der Wissenschaften zu Berlin* Ernst zu machen (Dubiel et al. 1986). Eine weitere HRG-Novellierung (1985) schraubte derweil – zwecks „Entbürokratisierung“ und „Autonomie“ (statt Mitbestimmung) – an der Ausdehnung der Drittmittelforschung (Schäfer 1985). Noch ein Brandanschlag wurde verübt, diesmal (im Rahmen der *BIG-TECH* 86) auf den Dienstwagen des Roboter-Professors Spur (SFB 57); und das vielbesuchte Gründerzentrum BIG, zwischenzeitlich um einen *Technologie- und Innovationspark* (TIP) erweitert, wurde Mitte 1987 privatisiert – auch dies, wie es hieß, ein Zeichen der Zeit.

Die „überzogenen Erwartungen“ an die Jungunternehmer waren da allerdings schon verpufft (Heine 1986: 64). Von einem „Mekka der Telekommunikation“ wäre trotz des vielen Innovationsklima-Aktionismus dann auch wenig zu sehen, resümierte das *Forum Wissenschaft* des Bund demokratischer Wissenschaftlerinnen und Wissenschaftler, „zumal unter den [resultierenden] Neugründungen Kioske und Würstchenbuden dominieren und der innovative Computerproduzent die absolute Ausnahme bildete“ (Lenz 1987: 116). Fröhlich gaben sich immerhin die „750 Jahre Berlin“-Feierlichkeiten: „Lösungen der Zukunft sind heute in der Stadt überall spürbar“, so Bundesforschungsminister Riesenhuber im Vorwort des Ausstellungskatalogs *Wissenschaften in Berlin*, wo einmal mehr am „Mythos Wissenschaftsstadt“ gefeilt wurde (vgl. AStA FU 1987).

**Figure 5:**
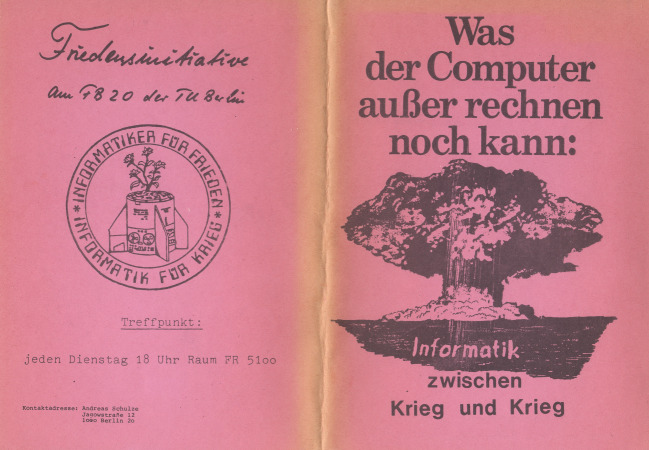

Die Konjunktur „kritischer“ Informatik ging mit solchen, vorwiegend lokalen Höhen und Tiefen freilich nicht ganz synchron. Andere, umfassendere Gefahren waren am Horizont aufgezogen und sorgten dafür, dass das „Umdenken in der Informatik“ für eine Zeit lang vom Randgruppengeschäft zur „berufsbezogenen“ Unternehmung mutierte. Insbesondere die im März 1983 von Reagan verkündete *Strategic Defense Initiative*, mit der simultan Kalter Krieg und US-amerikanische Hightech-Industriepolitik betrieben wurde, scheuchte (auch) BRD-Informatiker*innen auf (Slayton 2013; Stadler et al. 2020: V/22–V/29). Nicht mehr nur die „Korruption der Forschung“ standen damit zur Debatte, sondern *Game Over* für alle: „programmierter Atomkrieg“. Schon 1981 war am FB 20 eine Friedensinitiative ins Leben gerufen worden, „Treffpunkt: jeden Dienstag 18:00 Raum FR 5100“ (Anonym 1982c: 1) (Abb. 5).

Das Personal dieser Initiative, die mit den allerorts aufkeimenden Friedensbewegungen aufblühen sollte, wies, kaum überraschend, sehr deutliche Schnittmengen mit dem Projekt WILAB auf; gleiches gilt für die Berliner Regionalgruppe des im Juni 1984 in Bonn lancierten *Forums Informatiker für Frieden und gesellschaftliche Verantwortung* (FIfF). Das Forum, bis Anfang 1987 auf 13 Regionalgruppen und knapp 650 Mitglieder angewachsen, sollte „allen offen [stehen], die mithelfen wollen, die unselige Verbindung zwischen Informatik und Militär zu (zer-)stören“ (Kreowski 1984: 1). Hier ist beziehungsweise war nicht der Ort, diese Geschichte zu erzählen; unschwer ist aber ersichtlich, dass die Aussicht auf computerisierten Weltkrieg die Parameter informationstechnischen Gegenwissens über kurz oder lang in andere Bahnen lenkte. Nach wie vor schrieb sich das *Forum* (seit 1987: Forum InformatikerInnen) „sozialverträgliche EDV“ auf die Fahnen. Nach wie vor war am inhumanen Menschenbild der Informatik zu verzweifeln (die im Zuge von SDI verstärkt ins öffentliche Bewusstsein tretende „Künstliche Intelligenz“ galt tendenziell gar als dessen Apotheose). Überschattet wurde dieser Diskurs nun aber von den deterritorialisierten Gefahren spätmodern-atomarer Kriegsführung. Die Träume vom Wissen zum „örtlichen“ Gebrauch, von „Betroffenenwissenschaft“ und „Wirtschaftsdemokratie“ gerieten dabei latent unter die Räder; fast zwangsläufig erschöpfte sich der Einsatz für den planetaren Frieden in „destruktive[r] Kritik“ (Grosswiele 1986: 10).^19^

## Schluss

Die Spuren des WILAB, sowie die vieler Akteure überhaupt im Dunstkreis entsprechenden „Umdenkens“, verlieren sich irgendwann in den späten 1980er und frühen 1990er Jahren: Floyd kehrte der TU Berlin 1991 den Rücken, wo sich nun vollends eine „formal/technische Informatik-Sicht“ durchgesetzt hätte (Siefkes & et al. 1999: 141); die Herausgeber*innen der *Wechselwirkung* warfen 1989 das Handtuch (Stange 1990); Volpert, zunehmend isoliert, diagnostizierte eine anbrechende „Zeit […] des technozentrischen Denkens“ (Volpert 1994: 4). Die Ausnahme zur Regel, *Wuseltronick*: auf youtube findet sich ein Video, in dem der Kopf des Kollektivs, Reiner Lemoine, mit Gerhard Schröder durch Thalheim/Bitterfeld beziehungsweise „Solar Valley“ marschiert, dem Standort von (nunmehr) „Q-Cells“ (heute Hanwha Q Cells, mit Hauptsitz in Seoul) (vgl. Maron 2009). Irgendwann, „[z]wei bis vier Millionen Arbeitslose später“, sinnierte das Magazin *FIfF-Kommunikation* 1995, war „das Wort vom Jobkiller aus der Debatte“ verschwunden, trotz noch mehr Welt- und Binnenmarkt, trotz noch mehr Computer-im-Alltag (Bernhardt 1995: 3). Von Bewegung aber war nicht mehr viel zu spüren. „Die linken Kritiker der Informatik“, mittlerweile wieder zur Randerscheinung reduziert, „wollen nur noch das Schlimmste verhüten“, hieß es in einem anderen Abgesang, in der *konkret* (Fischer 1989: 12).

Immerhin. Für den eingangs angeschnittenen Verdacht, der da lautet: Komplizenschaft in punkto Schleifen des Elfenbeinturms, wird man sie (oder die WILA-„Bewegung“), jedenfalls kaum ernsthaft zur Verantwortung ziehen wollen. Nicht, dass es – „Gründerjahre“ – keine Resonanzflächen gegeben hätte. Wie gesehen, interagierte der zunächst unter sozialliberalen, dann neokonservativen Vorzeichen sich aufbauende Verwertungsdruck durchaus mit den diversen, in Richtung nützliches Wissen/nützliche Produkte tendierenden Initiativen „von unten“ – und sei es nur, wie im Fall BIG, um sich kritisch davon absetzen zu müssen. Das war in der Regel leichter gesagt als getan: kaum ein Weg etwa führte in punkto Hightech um die unschöne Tatsache herum, dass, wie der WILAB-„Reader“ *Computer in Alternativprojekten* (1983) zu bedenken gab, die „Bauteile der Mikroelektronik […] in Hongkong oder Korea von Frauen für Hungerlöhne“ hergestellt wurden (Anonym 1983a: 60). Ans „Aussteigen“ war so gesehen gar nicht erst zu denken: „Die ‚Alternativen‘ sind, soweit wir im Fach bleiben, Reformprojekte, selbstorganisierte Institute oder Betriebe wie die kleine ‚Computerklitsche‘“ (Anonym 1981: 6). Gerade weil die Übergänge hier durchaus fließend sein konnten – *Wuseltronick* etwa gingen mit ihren Produkten auf der *BIG-TECH* notgedrungen ebenso hausieren wie in der ‚Szene‘ – lohnt es sich also etwas genauer hinzuschauen. Zur Debatte standen hier offenbar nicht die Ausweitung von „Anwendungsorientierung“, die ja gewollt war (und wieso auch nicht), sondern deren strukturelle Bedingungen: die „Situation am Arbeitsplatz“ (Zeitverträge, sonstige Abhängigkeiten und Machtgefälle) (Anonym 1981: 6); die Konsequenzen des eingeschlagenen „Sparkurs“; das (jedenfalls so registrierte) „Abschotten der Wissenschaftsinstitutionen gegenüber alternativen Wissenschaftsinhalten“ (Rilling 1984: 36) im Zeichen von „Spitzenforschung“ und „Weltmarktkonkurrenz“. Umgekehrt, oder so ließe sich genauer zeigen, war allzu engagierte „Anwendung“ im universitären Bereich dann auch keinesfalls nur gerne gesehen: im Zweifelsfall hieß die Devise „Grundlagenforschung“, wie etwa das Zurückschrauben des BMFT-Programms „Humanisierung des Arbeitslebens“ in den 1980er Jahren nahelegt (vgl. etwa Kern 1981; Väth 1984). Die etwas unpräzise Rede vom Siegeszug (‚neoliberaler‘) Marktlogiken verdeckt derartige Dynamiken eher.^20^ Ähnlich wie der West-Berliner Sozialsenator Fink die Alternativwirtschaft vorwiegend in bestimmter Hinsicht umgarnen sollte – insofern sie, mit ein wenig „Staatsknete“ ausgestattet, den Rückbau von Sozialleistungen mitabfedern half –, so gestaltete sich auch der Weg aus dem Elfenbeinturm kaum ebenmäßig. Die „außerordentlich erwünschte Zusammenarbeit von Wissenschaft und Wirtschaft“ war das eine; „Augenmaß am Platz“ war allerdings beim Versuch geboten, so der seit 1982 amtierende Forschungsminister Riesenhuber, erstere zu *gesellschaftlichen *Zielsetzungen zu verpflichten (Der Bundesminister für Forschung und Technologie 1984: 20).

Im Rückblick erscheinen – Milchläden versus Brutstätten aus Beton – die damaligen Betriebe des Gegenwissens in jedem Fall weniger als Vorbereitung neoliberaler Wissens-Proprietisierung „von unten“, denn als Ausläufer verflossenen Reformwillens. So zumal legt es das hier ausgebreitete West-Berliner Szenario nahe. Ähnlich unplausibel wäre es wohl, derartige Unternehmungen als Keimzellen von Relativismus und romantischer Fortschrittsfeindlichkeit hochzustilisieren. Über andere, gerade Akademie-fernere Milieus und deren womöglich längerer Atem ist damit freilich zwar nichts gesagt; nicht ganz abseitig aber ist die Vermutung, dass die besagte „Abschottung“ der Forschung gegenüber „alternativen“, in der Regel nämlich gesellschaftlichen Zielsetzungen in punkto Experten- und Wissenschaftsskepsis etwas mehr ins Gewicht fallen dürfte, als die derzeitige, vor allem auf Protest-Phänomene geeichte Diskussion dies vermuten lässt.^21^ Wie „Greedy Science“ à la BRD funktioniert(e), um hier einen kürzlich in Umlauf gebrachten Slogan aufzunehmen, sollte die hiesige Wissenschaftsgeschichte so gesehen mindestens genauso beschäftigen, wie die Widerstände, die dies unweigerlich auslöste.^22^ Bislang kommunizieren die entsprechenden historiographischen Genres wenig bis kaum: jene, die sich mit den Weichenstellungen „von oben“ beschäftigen; und solche, die vor allem die Gegenmilieus im Blick haben, nicht immer aber den Gegenstand des Dagegenseins.^23^

## Footnotes

1 So etwa (aus holländischer Perspektive): „We wanted a university more involved with society […] but we got a university that has reduced ‚society‘ to ‚business‘“ (Halffman & Radder 2015: 173). Zur BRD, siehe Block-Künzler & Graf 1993.

2 Zu diesem Topos vgl. etwa Dean & Zamora (2021); Falasca et al. (2018); Neumann (2008). Zur Kritik daran, mit der hier sympathisiert wird, vgl. Neuheiser (2017); sowie allgemeiner z. B. Baker (2022); Cooper (2017).

3 Die Diagnose – das „Irrationale als die neue Moral“ u. ä. – wurde damals zwar nicht nur, aber auch nicht zuletzt von konservativer Seite bemüht, siehe etwa Noelle-Neumann & Maier-Leibnitz (1987).

4 Einen aktuellen Überblick bieten Leendertz (2022); Szöllösi-Janze (2021).

5 Siehe z. B. Häberlen (2018); Hannig (2021); Müller (2020); umfassend zum „linksalternativen Leben“ in der BRD, siehe Reichardt (2014).

6 Einen Überblick bzgl. „Transfer“ bieten Meier & Krücken (2010); zu „Innovation“ vgl. Weber (2018).

7 Zu den „Betroffenen“ zählten, wenig überraschend, v. a. Frauen und „Ausländer“, dazu siehe Zeppenfeld (2021). Vgl. ferner zu Wolfsburg/Wilhelmshaven bzw. Hamburg: Eiben (2019); Neumann (2018).

8 Zur Forschungspolitik der 1970er Jahre vgl. etwa Abelshauser (2009): IV.

9 Der Sektor „Bildung und Wissenschaft“ wuchs von ca. 30.000 (1970) auf über 50.000 (1982) Beschäftige an – „beinahe die Hälfte der gesamten Neueinstellungen“ im ÖTV (DGB Landesbezirk Berlin 1984: 78).

10 Volker Hauffs (BMFT) Koautor, Fritz W. Scharpf, war seit 1973 Direktor am WZB.

11 Einen Überblick bieten: Burmeister & Canzler (1988).

12 Selbst der ominöse SFB 57 sollte hier seinen Beitrag leisten: dessen Direktor Günter Spur dürfte Wissenschaftshistoriker*innen v. a. als Historiograph des Berliner Maschinenbaus bzw. der „Psychotechnik“ bekannt sein. Vgl. u. a. Spur (1979).

13 Siehe Henschel-Fotobestand, Signatur: B01_0131_12-20, FHXB-Museum, https://fhxb-museum.de/fhxb_lp/objekt.php?o=21097&m=37473 (18.05.2022).

14 Kritik an derartigem „Technologietransferaktionismus“ übten denn auch nicht zuletzt neoliberal veranlagte Zeitgenossen bzw. Ökonomen, vgl. etwa Staudt (1985).

15 So der Tenor der damaligen „Neo“-Schumpeterianischen Diagnosen. Zentraler Akteur hierbei war, wie erwähnt, Mensch (WZB), auf den sich Glotz und Allesch, der Leiter von *TU-transfer*, regelmäßig beriefen.

16 Zur „flächendeckenden Etablierung von universitären Transferstellen“ in den 1980er Jahren siehe Meier & Krücken (2010).

17 Dazu musste man natürlich nicht unbedingt einen Wissenschaftsladen eröffnen. Zum damaligen (tendenziell gewerkschaftsnahen) Kontext vgl. etwa Briefs et al. (1983).

18 Teilweise wurde man diesbezüglich auch von der Industrie überholt: vgl. Asaro (2000). An Selbstkritik fehlte es zunehmend ebenfalls nicht: Die Ansätze wären evident „unwirtschaftlich“ und überforderten die „Betroffenen“, hieß es etwa.

19 Zur etwas anders gelagerten Trajektorie der US-Schwesterorganisation *Computer Scientists for Social Responsibility* vgl. Finn & DuPont (2020).

20 Weiterführend siehe Agar (2019: 94–99); Popp Berman (2013); Stadler & Wulz (2020).

21 Zu Überlegungen, die in diese Richtung zielen vgl. Shapin (2019).

22 Siehe https://greedyscience.princeton.edu/ (18.05.2022).

23 Dass die Kategorien oben/unten diesbezüglich nur bedingt tragfähig sein dürften, versteht sich von selbst; vgl. dazu Mody (2022).
